# Multimodal Spatiotemporal Deep Learning Framework to Predict Response of Breast Cancer to Neoadjuvant Systemic Therapy

**DOI:** 10.3390/diagnostics13132251

**Published:** 2023-07-03

**Authors:** Monu Verma, Leila Abdelrahman, Fernando Collado-Mesa, Mohamed Abdel-Mottaleb

**Affiliations:** 1 Department of Electrical and Computer Engineering, University of Miami, Miami, FL 33146, USA; monuverma.cv@gmail.com; 2 MIT Media Lab, Cambridge, MA 02139-4307, USA; leilaabd@media.mit.edu; 3 Department of Radiology, Miller School of Medicine, University of Miami, Miami, FL 33146, USA; fcollado@med.miami.edu

**Keywords:** multimodal deep learning framework, automated neoadjuvant systematic therapy prediction, 3D-CNN multimodal framework

## Abstract

Current approaches to breast cancer therapy include neoadjuvant systemic therapy (NST). The efficacy of NST is measured by pathologic complete response (pCR). A patient who attains pCR has significantly enhanced disease-free survival progress. The accurate prediction of pCR in response to a given treatment regimen could increase the likelihood of achieving pCR and prevent toxicities caused by treatments that are not effective. Th early prediction of response to NST can increase the likelihood of survival and help with decisions regarding breast-conserving surgery. An automated NST prediction framework that is able to precisely predict which patient undergoing NST will achieve a pathological complete response (pCR) at an early stage of treatment is needed. Here, we propose an end-to-end efficient multimodal spatiotemporal deep learning framework (deep-NST) framework to predict the outcome of NST prior or at an early stage of treatment. The deep-NST model incorporates imaging data captured at different timestamps of NST regimens, a tumor’s molecular data, and a patient’s demographic data. The efficacy of the proposed work is validated on the publicly available ISPY-1 dataset, in terms of accuracy, area under the curve (AUC), and computational complexity. In addition, seven ablation experiments were carried out to evaluate the impact of each design module in the proposed work. The experimental results show that the proposed framework performs significantly better than other recent methods.

## 1. Introduction

In the U.S., about 1 in 8 women (13%) are expected to develop invasive breast cancer during their life. Breast cancer is the most diagnosed cancer among U.S. women, accounting for an estimated 30% of newly diagnosed cancers, and breast cancer ranks second amongst the leading causes of cancer-related death in women in the U.S. [1]. Current approaches to breast cancer therapy include neoadjuvant systemic therapy (NST), which has several potential advantages including avoiding mastectomy by reduction in tumor size and downstaging the axilla, which may obviate the need for axillary lymph node dissection and its potential complications. In addition, NST also permits the in vivo assessment of drug efficacy with the possibility to opt for a different treatment approach if the tumor is not responding. The efficacy of NST is measured by pathologic complete response (pCR). A patient who attains pCR has significantly enhanced disease-free progress survival. The accurate prediction of which patients will achieve pCR in response to a given treatment regimen could increase the likelihood of achieving pCR and prevent toxicities caused by treatments that are not effective [2].

The response to NST is traditionally assessed by a combination of physical exams and dedicated breast imaging exams, most commonly dynamic contrast enhance magnetic resonance imaging (DCE-MRI). Radiologists typically assess this response by measuring changes across a limited handful of clinical imaging parameters on DCE-MRI over the course of treatment, such as a tumor’s largest diameter (LD) or its pattern of contrast agent uptake. More recently, hand-crafted radiomic imaging features [3,4,5,6] have been shown to predict pCR from pre-treatment DCE-MRI data by characterizing the texture of the tumor and micro-environment. Nonetheless, radiomics assessment is limited by the need to pre-define meaningful and predictive hand-crafted features to assess the attributes such as lesion shape and image texture, and radio mic models are constrained by the discriminability of a finite and pre-defined pool of features.

Recent advancements in artificial intelligence (AI), especially deep learning (DL) [7,8,9], have shown a significant improvement compared to the conventional approaches [10,11] in the prediction of pathological results through radiological data. DL has become the most effective technique for various applications such as prediction, classification, object detection, and image segmentation. DL makes it possible to automatically extract features from MRI exams instead of engineered features, and it achieves impressive performance for the prediction of the response to NST [7,8]. Data obtained from tumor samples through biopsies can also shed light on cellular biomarkers such as HER2, ER, and PgR. These biomarkers can help guide clinicians’ choices on which NST to prescribe. For example, a patient with ER-positive cancer may respond well to Tamoxifen, a hormone therapy that targets ER-positive cells. On the other hand, if a patient is ER-negative, they will fail to respond to this drug and may require chemotherapy. Combining information from different modalities, e.g., MRI exams and tumor pathological features can help with the development of robust machine/DL algorithms to help predict the response to NST, as shown by some researchers. For example, Ravichandran et al. [7] introduced a deep learning-based unique patch-based response prediction approach that allows for the visualization of specific spatial regions and image patterns associated with poor or favorable responses to NST. They introduced a CNN model to learn the features from the pre- and post-contrast pre-treatment imaging and yielded an AUC of 0.77. In addition, they also validated that the incorporation of the HER2 biomarker features with MRI could yield an AUC of 0.85. Duanmu et al. [12] further showed that convolving the clinical features with the imaging features learned in the CNN instead of concatenating them could yield an accuracy of 0.83 and an AUC of 0.80. However, these studies considered only pre-NST MRI data along with clinical reports. Therefore, these approaches neglected the structural and functional tumor changes on DCI-MRI over the course of NST. The structural and functional changes in the tumor microenvironment at different stages of the NST regimen can better reflect the therapeutic response [13,14].

We propose a multimodal spatiotemporal deep learning framework by incorporating multimodal information for predicting the response of breast cancer to neoadjuvant therapy. The proposed work has the following key contributions:

1. We develop a multimodal spatiotemporal deep learning by integrating the following multi-modalities: imaging data with N-time stamps (pre-treatment, early treatment, inter-regimen, prior to surgery, etc.), molecular data (ER, PgRPos, HRPos, BilateralCa, Laterality, HER2Pos, HR_HER2_Category, and HR_HER2_Status), and demographical data (age and race). We demonstrate the influence of each time point on the predictions made by the network through ablation experiments.
2. We design a novel 3D-CNN-based deep learning framework by introducing a cross-kernel feature fusion (CKFF) module.
3. The CKFF module makes the architecture more learnable at a lower computational cost by paying attention to multiple receptive fields to extract the spatiotemporal features.
4. The efficacy of the proposed framework is tested on a challenging breast cancer data set, ISPY-1 [15], in terms of accuracy and AUC.

## 2. Methodology

In computer vision applications, the conventional CNN models VGG 16-Net [16], VGG19-Net [16], and ResNet [17] have demonstrated impressive results. On the other hand, a deep, dense network prevents these models from preserving important aspects of breast cancer MRI scans. Progressive convolution and pooling operations may cause the deep, dense CNN architectures to overlook the cancerous regions’ micro-level features [18]. In addition, large data samples are required for deep dense CNN architectures to learn important features. Profound CNN networks fail to learn appropriate features over smaller datasets and endure overfitting [19,20]. Only a few samples in the benchmark datasets of NST are available. Recently, Qu et al. [14] and Ravichandran et al. [7] proposed a deep learning-based solution for NST prediction and overcame the issue of limited data samples through data augmentation. Additionally, before supplying images to the network, they used segmentation. Huynh et al. [21] also found a solution to the problem of overfitting by combinining the LDA classifier with the transfer learning capabilities of VGGNet. However, its two-stage framework makes the presented strategy challenging to implement in real-time applications. We proposed an end-to-end multimodal spatitemporal deep learning framework to extract spatiotemporal features from the MRI scans to predict the pCR response of neoadjuvant treatment.

### Proposed Method

**Figure 1:**
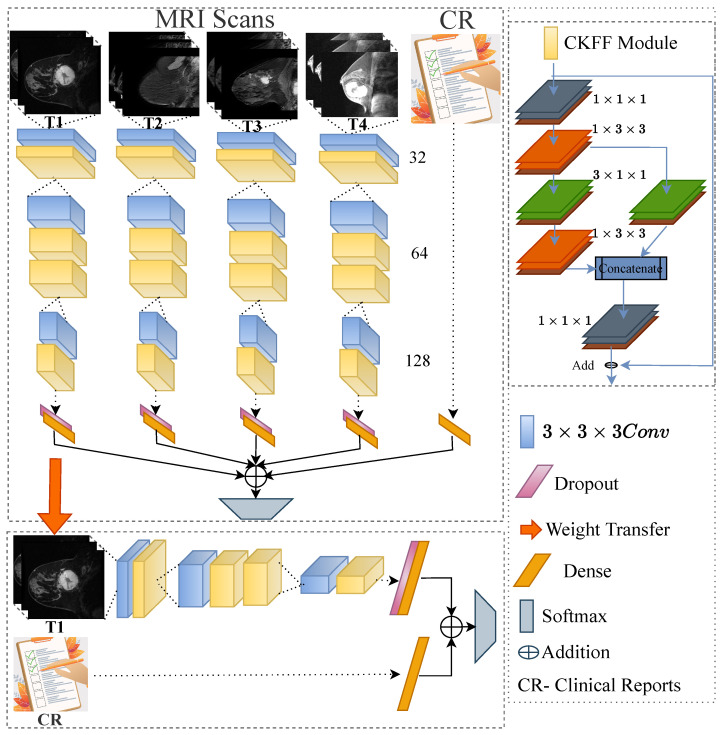
The proposed deep-NST prediction framework architecture. Image sequences with different time stamps (represented by $T_{1}$, $T_{2}$, $T_{3}$, and $T_{4}$) are processed through parallel 3D-CNN networks. The feature outputs from all time stamps and clinical reports are added and processed by a final soft-max layer. Further trained weights of the first 3D-CNN network are fine-tuned over image sequences captured at the early stage of the treatment ($T_{1}$) along with the clinical features. The numbers in the image denote the size of the feature dimension at each layer.

The proposed multimodal spatiotemporal deep learning framework comprises four parallel 3D-CNN networks along with clinical features, as shown in Figure 1. Specifically, the 3D-CNN network is introduced to learn spatial and temporal features from the MRI scans at a particular time-stamp. In addition, parallel 3D-CNN networks are used to learn the structural and functional changes in the tumor microenvironment at four-time stamps $T_{1}$, $T_{2}$, $T_{3}$ and $T_{4}$, as shown in Figure 1. Further, clinical reports’ features, including molecular and demographical data, are processed to aid imaging features to enhance the generalization of the deep-NST framework. Moreover, we used the LeakyReLU as an activation function over the resultant features of each convolution layer.

The proposed deep-NST framework is trained by using two stages to predict the outcome of the NST, as shown in Figure 1. The proposed deep-NST framework is initially trained using MRI scans captured at four-time stamps ($T_{1}$, $T_{2}$, $T_{3}$, and $T_{4}$) and clinical data. Further, knowledge of the 1st 3D-CNN network associated with early-stage MRI scans ($T_{1}$) is finetuned again with $T_{1}$ MRI scans and clinical features. The motivation behind developing this framework was to utilize the multimodal spatiotemporal features of different time stamps along with clinical data to train deep learning methods. However, the final prediction model only needed pre-NST MRI scans with clinical data and ensured early-stage prediction for NST in breast cancer.

## 3. Experimental Results and Analysis

In this section, first we present the dataset and the implementation details. Further, the experimental setups and experimental results are discussed. In addition, we explore the importance of each module of the proposed framework in the ablation study. Finally, we compare the computational complexity of the proposed model with state-of-the-art approaches.

### 3.1. Dataset

**Figure 2:**
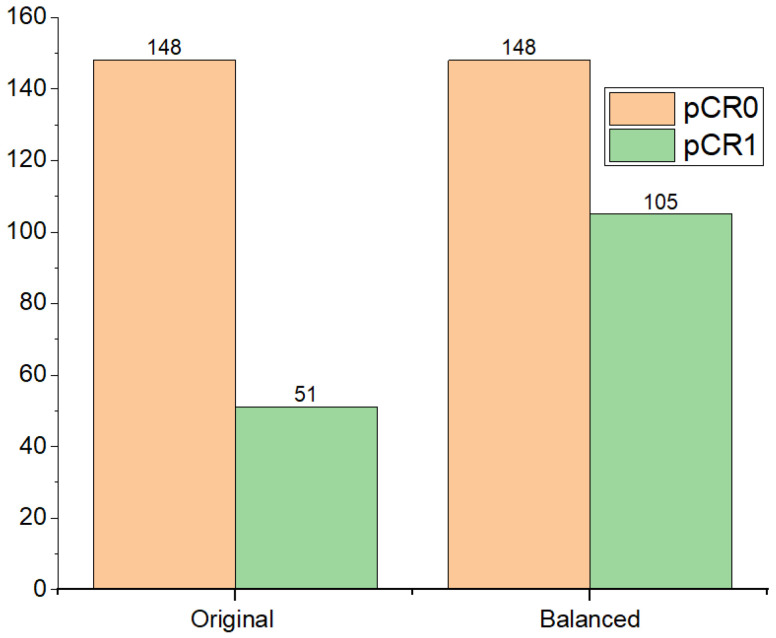
Data sample distribution in pCR0 and pCR1 classes of ISPY-1. In the original dataset, each patient was represented by a single instance of MRI scans. However, in the balanced dataset, we have made updates to include multiple MRI scans at the same time-stamp for patients who responded to the NST.

To validate the effectiveness of the proposed deep-NST framework, we work with the ISPY-1 [15] public dataset’s dynamic contrast-enhanced (DCE)-MRI images and non-imaging clinical report information. The ISPY-1 dataset comprises a cohort of 207 patients, out of which only 121 had MRI scans at all four-time points (pre-treatment, early treatment, inter-regimen, and prior to surgery). The original dataset exhibited a significant disparity in class distribution, with the majority of samples belonging to the non-responded (pCR0) class, as shown in Figure 2. We noticed instances where certain patients underwent multiple MRI scans at the same timestamp. To address the class imbalance and achieve a balanced distribution for both classes, i.e., non-responded (pCR0) and responded (pCR1) to NST, we included multiple MRI scans captured at the same timestamp for the responding class as shown in Figure 2. However, for the non-responding class, we considered only a single MRI scan. Ultimately, we collected a total of 148 samples from 84 patients in the non-responding class and 105 samples from 37 patients in the responding class for each timestamp. Additionally, we utilized ten non-imaging clinical features (age, race, ERPos, HRPos, PgRpos, Her2MostPos, HR_HER2_CATEGORY, HR_HER2_STATUS, BilaterCa, and Laterality) from the clinical data dictionary of the ISPY-1 trial. In this study, we focused on 121 patients from the ISPY-1 trial, splitting them into a training set consisting of 84 patients with 182 data samples, and a testing set comprising 37 patients with 71 data samples.

### 3.2. Training and Implementation Details

We used two stages to train the proposed deep-NST framework: training with four-time stamps NST MRI scans and clinical data and fine-tuning with only pre-NST MRI scans and clinical data, as shown in Figure 1. The first stage is trained for 300 epochs, whereas the second stage is finetuned by using 50 epochs. Moreover, final testing is done end-to-end by utilizing pre-treatment NST MRI scans and clinical data captured at the initial stage of the treatment to ensure the early-stage prediction for NST in breast cancer.

The publicly available ISPY-1 breast cancer dataset has MRI scans with different durations. To overcome this issue, we use the temporal interpolation model (TIM) [22] to normalize the length of the scan sequences to 60. The image sequences are normalized to 112×112×60 before using a spatiotemporal CNN model. To ensure a fair comparison of CNN-based networks, we implemented the conventional deep learning models: VGGNet [16], and ResNet with 3D-CNN [17], and trained them over our experimental settings. Moreover, we tested the effects of cross-entropy loss function over focal loss [23] by evaluating the results of the proposed deep-NST with focal loss and named deep-NST+Focal. All implementations use python 3.6 with Keras 2.3.1 and Tensorflow 2.1.0. We utilized the SGD optimizer for training the models with a 0.001 learning rate, 0.9 momentum, and 0.01 weight decay. The cross-entropy loss function is used for network optimization.

### 3.3. Experimental Results Analysis

This section presents the experimental results of ISPY-1. We used the prediction accuracy and AUC-ROC performance measures to evaluate the proposed deep-NST with state-of-the-art NST approaches. The AUC-ROC curve represents the degree or measure of separability. The AUC-ROC measures the capability of the model to distinguish between classes by analyzing the true positive response (*TPR*) against the false-positive response (*FPR*). The *TPR* and *FPR* are calculated by using Equations (1) and (2).

(1) $$TPR=\frac{True\;Positive}{Total\;no.\;of\;data\;samples}$$

(2) $$FPR=\frac{Flase\;Positive}{Total\;no.\;of\;data\;samples}$$

Moreover, the prediction accuracy is calculated by the following Equation (3).

(3) $$ACC=\frac{Total\;no.\;of\;correctly\;predicted\;samples}{Total\;no.\;of\;data\;samples}$$

**Figure 3:**
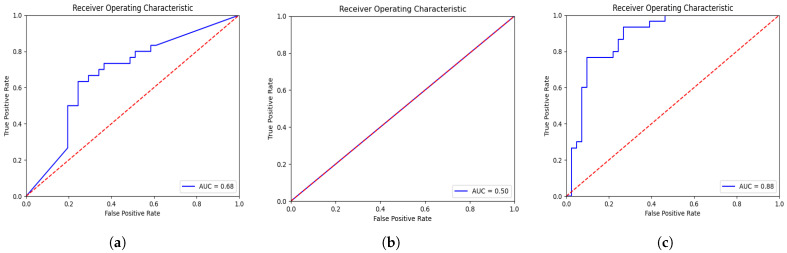
ROC curves for pCR prediction models on the ISPY-1 dataset: (**a**) conventional 3D-VGGNet (AUC = 0.68), (**b**) conventional 3D-ResNet (AUC = 0.50), and (**c**) proposed deep-NST (AUC = 0.88).

**Figure 4:**
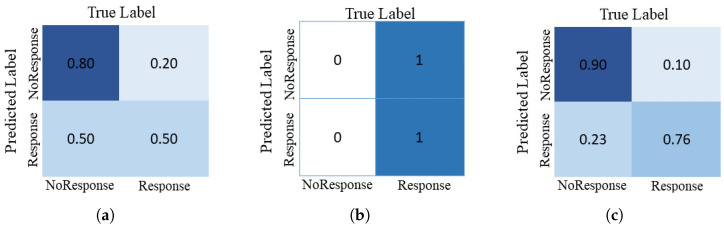
Confusion matrices of (**a**) 3D-VGGNet, (**b**) 3D-ResNet, and (**c**) proposed deep-NST frameworks.

The evaluation results for proposed and conventional CNN models are reported in Table 1. From Table 1, it is validated that the proposed deep-NST framework gains significantly better performance than traditional as well as the current deep learning models for NST. Particularly, the proposed deep-NST achieves 0.20% and 0.38% more AUC than the 3D-VGGNet and 3D-ResNet CNN models, respectively. More detailed results in terms of ROC for 3D-VGGNet, 3D-ResNet, and proposed deep-NST are illustrated in Figure 3. In addition, for detailed class generalization, we have calculated the confusion matrices as shown in Figure 4. From Figure 4b, it is clear that the 3D-ResNet is under-fitted compared to the data and is not suitable for the NST due to limited data samples. In, addition, when we observed the confusion matrices for the existing 3D-VggNet (Figure 4a) and proposed deep-NST (Figure 4b), we saw a skew towards high accuracy in predicting the non-responder patients over responder patients. This may be due to the original skewed dataset distribution. In addition to accuracy and AUC, we also evaluated sensitivity (0.9024) and specificity (0.7666). Sensitivity tells us the proportion of true positives that the model correctly identified. It gives us an idea of how well the model detects the condition when it is actually present. On the other hand, specificity measures the proportion of true negatives that the model correctly identified. It helps us understand how well the model identifies the absence of the condition.

#### 3.3.1. Discussion

In contrast to other methods, we highlight a reduced reliance on feature engineering and manual segmentation (lesion segmentation) during image preprocessing to yield comparable results to current state-of-the-art methods. For example, authors [4,5,6] rely on precalculating the tumor volume or functional tumor volume as a feature before being input into a machine learning classifier. This extra preprocessing step is costly. We rely only on the raw DCE-MRI images and let the network learn the relevant image features through back-propagation. Even compared to existing end-to-end DL methods [7,12,16,17], AUC (0.88) outperforms the prior results. Moreover, our approach uses imaging data from all four-time points in a patient’s NST regimens, which allows the proposed deep-NST to outperform or match the current DL state-of-the-art methods [7,12,24] that rely on data from the only one-time point.

#### 3.3.2. Ablation Study

We have conducted seven ablation experiments to evaluate the importance of each component step-by-step in the proposed deep-NST. First, the CKFF module’s effect is tested by comparing it to the basic 3D convolution layer. The proposed CKFF module is replaced by the 3D convolution of size 3×3×3 and named deep-NST+3DCNN. The second study has been conducted to examine the CKFF module’s impact over parallel multi-scale convolutional layers and is named deep-NST+3DInception. The other study has been conducted to test the effect of multimodal information on clinical data and MRI data. For this study, we used the information from the MRI imaging and called it a Unimodal ST. The results of Unimodal ST are computed on imaging data only. The comparative results for proposed deep-NST and ablation studies are tabulated in Table 1 and Table 2. The results show that the cross-entropy loss function, the proposed CKFF module, and multimodal data play an important role in the deep-NST framework. Specifically, the proposed framework has 0.23%, 0.23%, and 0.01%, higher AUC than deep-NST+3DCNN, deep-NST+3DInception, and Unimodal ST. Similarly, the proposed framework gain 0.19%, 0.19%, and 0.02% higher accuracy over deep-NST+3DCNN, deep-NST+3DInception, and Unimodal ST.

**Figure 5:**
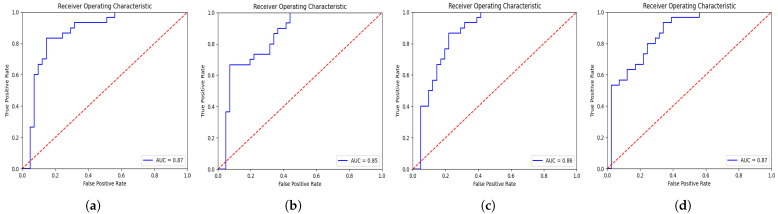
ROC curves for the proposed model on different inputs: (**a**) only $T_{1}$ MRI scans; (**b**) $T_{1}$ MRI scans with clinical information; (**c**) $T_{1}$ and $T_{2}$ MRI scans with clinical information; and (**d**) $T_{1}$, $T_{2}$, and $T_{3}$ MRI scans with clinical information, over ISPY-1 dataset.

**Figure 6:**
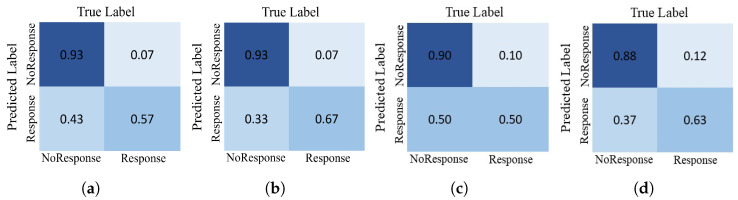
Confusion matrices of the proposed model for different inputs: (**a**) only T1 MRI scans; (**b**) $T_{1}$ MRI scans with clinical information; (**c**) $T_{1}$ and $T_{2}$ MRI scans with clinical information; and (**d**) $T_{1}$, $T_{2}$, and $T_{3}$ MRI scans with clinical information, over ISPY-1 dataset.

Moreover, we also investigated the effect of each timestamp MRI scan with the clinical information. The experimental results are tabulated in Table 2. The results show that combining all time stamps’ MRI scans with clinical information allows the network to learn the pertinent features of the breast cancer image sequences and achieve better performance in predicting the outcome of NST. More detailed results in terms of ROC and class-wise confusion matrix are illustrated in Figure 5 and Figure 6, respectively. This figure shows an ablation study by including the time instances during the neoadjuvant regimen (inter-regimen), gradually increasing the model’s performance.

#### 3.3.3. Complexity Analysis

This section represents the comparative study between the traditional 3D-CNN, proposed deep-NST, and its variants: deep-NST+3DCNN, deep-NST+3dInception, and Unimodal ST (spatiotemporal) frameworks in terms of computational complexity. The total number of parameters engaged in each network is presented in Table 3. The proposed deep-NST has significantly fewer learnable parameters; 4 million (M) fewer than other existing NST frameworks such as 3D-VGGNet; 225M, and 3D-ResNet; 311M. Furthermore, deep-NST requires only 37 megabytes (MB) of memory storage, which is minor compared to 3D-VGGNet; 1.8 gigabytes (GB) and 3D-ResNet; 2.5 GB. Additionally, the total number of floating-point operations is much less for the proposed deep-NST than for the 3D-VGGNet and 3D-ResNet models. Moreover, the proposed deep-NST requires 17M fewer parameters than the deep-NST with a parallel multi-scale inception module. Similarly, the proposed deep-NST needs 1363 MB less memory than the deep-NST with a parallel multi-scale inception module.

This validates that the proposed CKFF module is cost-effective. Based on the experimental results and the computational complexity reported in Table 1 and Table 3, respectively, we can conclude that the proposed deep-NST is the most effective and efficient DL-based model for NST outcome prediction.

## 4. Conclusions

We present a compact and lightweight multimodal spatiotemporal deep learning framework to predict breast cancer response to neoadjuvant therapy by incorporating patients’ MRI, tumor molecular, and demographics features. MRI exams were obtained at different stages of treatment. The proposed framework performs better than state-of-the-art NST approaches. In addition, based on the conducted computational cost analysis, our framework is cost-effective based on the number of parameters and FLOPS compared to other models.

## Figures and Tables

**Table 1:** The AUC score of various methods using the ISPY-1 dataset. Results above the midline are feature-engineering methods. On the other hand, results below the line are DL-based.

Method	AUC	ACC
Volume [4]	0.73	N/A
FTV [5]	0.73	N/A
FTV and Varying PER and SER [6]	***0.90***	N/A
CNN and Feature Convolution [12]	0.80	N/A
CNN pre-post contrast [7]	0.85	N/A
3D-VGGNet * [16]	0.68	0.68
3D-ResNet * [17]	0.50	0.42
Deep-NST+Focal	0.88	0.79
Deep-NST+3DCNN	0.60	0.58
Deep-NST+3DInception	0.61	0.58
UniModal ST	0.84	0.72
**Deep-NST**	**0.88**	**0.85**

N/A indicates the information was not available. Here, * with 3D VGGNet and 3D ResNet represents that these models are trained from scratch.

**Table 2:** The AUC and accuracy score for proposed framework and ablation studies using different inputs of the ISPY-1 dataset.

Input	AUC	ACC
$T_{1}$ MRI Scans	0.87	0.77
$T_{1}$ MRI Scans + Clinical Data	0.85	0.82
$T_{1}$ + $T_{2}$ MRI Scans + Clinical Data	0.86	0.73
$T_{1}$ + $T_{2}$ + $T_{3}$ MRI Scans + Clinical Data	0.87	0.77
**$T_{1}$ + $T_{2}$ + $T_{3}$ + $T_{4}$ MRI Scans + Clinical Data**	**0.88**	**0.85**

**Table 3:** The computational complexity analysis for existing and proposed spatiotemporal deep learning framework.

Method	#Param.	#Mem	#FLOPS
3D-VGGNet [16]	225 MB	1.8 GB	$5.52\times 10^{3}$ G
3D-ResNet [17]	311.0 MB	2.5 GB	$1.95\times 10^{2}$ G
Deep-NST+Focal	4 MB	37.0 MB	30.5 G
Deep-NST+3DCNN	3.8 MB	31.3 MB	2.85 G
Deep-NST+3DInception	171.0 MB	1.4 GB	$1.49\times 10^{3}$ G
Unimodal ST	4 MB	36.6 MB	30.5 G
Deep-NST	4 MB	37.0 MB	30.5 G

Here, 3DInception is the 3D convolutional inception module [25,26].

## Data Availability

Data is unavailable.
